# Neonatal resuscitation monitoring: A low-cost video recording setup for quality improvement in the delivery room at the resuscitation table

**DOI:** 10.3389/fped.2022.952489

**Published:** 2022-11-02

**Authors:** Linus Olson, Xuan Anh Bui, Allan Mpamize, Hien Vu, Jolly Nankunda, Tung Thanh Truong, Josaphat Byamugisha, Tina Dempsey, Clare Lubulwa, Axel Winroth, Daniel Helldén, NeoSupra & NeoSpirit Teams, Anh Duy Nguyen, Tobias Alfvén, Nicolas Pejovic, Susanna Myrnerts Höök

**Affiliations:** ^1^ Department of Women’s and Children’s Health, Karolinska Institutet, Stockholm, Sweden; ^2^ Training and Research Academic Collaboration (TRAC) Sweden - Vietnam, Hanoi, Vietnam; ^3^ Neonatal Department, Vietnam National Children’s Hospital, Hanoi, Vietnam; ^4^ Department of Global Public Health, Karolinska Institutet, Stockholm, Sweden; ^5^ Department of Information Technology, Phu San Hanoi Hospital, Hanoi, Vietnam; ^6^ Dr. Ronald Batte Hospital, Entebbe, Uganda; ^7^ Social Department, Phu San Hanoi, Hanoi Obstetrics and Gynecology Hospital, Hanoi, Vietnam; ^8^ Department of International Collaboration, Phu San Hanoi Hospital, Hanoi, Vietnam; ^9^ Mulago Specialized Women and Neonatal Hospital, Kampala, Uganda; ^10^ Department of Pediatrics and Child Health, College of Health Sciences, Makerere University, Kampal, Uganda; ^11^ Department of Obstetrics and Gynaecology, College of Health Sciences, Makerere University, Makerere, Uganda; ^12^ Astrid Lindgren Children's Hospital, Karolinska University Hospital, Solna, Sweden; ^13^ Department of Medicine Huddinge, Center for Hematology and Regenerative Medicine, Karolinska Institutet, Stockholm, Sweden; ^14^ Department of Hospital Administration, Phu San Hanoi Hospital, Hanoi, Vietnam; ^15^ Sachs’ Children and Youth Hospital, Stockholm, Sweden; ^16^ Centre for International Health, University of Bergen, Bergen, Norway

**Keywords:** video camera, NeoTap, NeoBeat, neonatal resuscitation, neonatal diagnostics, video recording, quality improvement

## Abstract

**Background:**

The quality of neonatal resuscitation after delivery needs to be improved to reach the Sustainable Development Goals 3.2 (reducing neonatal deaths to <12/1,000 live newborns) by the year 2030. Studies have emphasized the importance of correctly performing the basic steps of resuscitation including stimulation, heart rate assessment, ventilation, and thermal control. Recordings with video cameras have previously been shown to be one way to identify performance practices during neonatal resuscitation.

**Methods:**

A description of a low-cost delivery room set up for video recording of neonatal resuscitation. The technical setup includes rechargeable high-definition cameras with two-way audio, NeoBeat heart rate monitors, and the NeoTapAS data collection tools for iPad with direct data export of data for statistical analysis. The setup was field tested at Mulago National Referral Hospital, Kampala, Uganda, and Phu San Hanoi Hospital, Hanoi, Vietnam.

**Results:**

The setup provided highly detailed resuscitation video footage including data on procedures and team performance, heart rate monitoring, and clinical assessment of the neonate. The data were analyzed with the free-of-charge NeoTapAS for iPad, which allowed fast and accurate registration of all resuscitative events. All events were automatically registered and exported to R statistical software for further analysis.

**Conclusions:**

Video analysis of neonatal resuscitation is an emerging quality assurance tool with the potential to improve neonatal resuscitation outcomes. Our methodology and technical setup are well adapted for low- and lower-middle-income countries settings where improving neonatal resuscitation outcomes is crucial. This delivery room video recording setup also included two-way audio communication that potentially could be implemented in day-to-day practice or used with remote teleconsultants.

## Introduction

The quality of neonatal resuscitation after delivery needs to be improved. In 2020 approximately 6,700 newborns died globally every day, 47% of all child deaths before 5 years of age. Most of these deaths occur during delivery, and the first day of life, and 98% in low- and middle-income countries (LMIC) (1, 2).

The Sustainable Development Goals (SDG 3.2) includes a specific target to end preventable neonatal deaths and decrease neonatal mortality to under 12 per 1,000 live births in all countries by 2030 (3). The most important causes of neonatal mortality are complications associated with preterm birth, birth asphyxia, or infections (4, 5). Birth asphyxia, and failure to initiate spontaneous breathing at birth, need early intervention, including warming, drying, and stimulation of the neonate. 3%–6% of all neonates need respiratory support with positive pressure ventilation (PPV) as part of the initial resuscitation (6). Delaying PPV leads to a progressive decrease in oxygenation and heart rate and potential death and/or brain injury in surviving neonates (7–10). Ventilation, early assessment of heart rate, and basic steps such as drying, and stimulation have been emphasized (11–16).

To improve neonatal resuscitation, we need to recognize what happens during this crucial time in a newborn's life, so we can review and optimize neonatal resuscitation to reduce unnecessary morbidity and mortality. This is true globally, from low- to high-resource settings. Furthermore, this is crucial in day-to-day services and clinical studies. It is essential to study neonatal asphyxia and its management in clinical settings. The need for basic and advanced resuscitation training and feedback for healthcare professionals, including doctors, midwives, and nurses, as well as teams, has been raised (17). A well-trained staff is key in all settings to reduce neonatal mortality (18).

We introduce a low-cost, still high-quality way of identifying performance practices during neonatal resuscitation using a video camera, heart rate meter (NeoBeat, Laerdal Global Health, Stavanger, Norway), and high-performance analyzing equipment (NeoTapAdvancedSupport, NeoTapAS, tap4life.org). The collected data can be used to improve resuscitation performance, including adherence to guidelines and closed-loop communication.

Our research team has used video cameras in previous studies and trials in Uganda, (19, 20) and others in the delivery room (21, 22). The NeoBeat dry-electrode ECG device is well adapted for LMIC where heart rate monitoring with a stethoscope is often not performed due to a lack of trained staff, stethoscopes, and pulse oximeters (23). The sensors pick up ECG-based signals recommended by The International Liaison Committee on Resuscitation (ILCOR) 2020 (7, 10) as the most reliable way to measure newborn heart rate (24). NeoTapAS for iPad was developed by members of our research group to register events during neonatal resuscitations and has been evaluated by our team and others (17, 25–28).

In this methodology article, we introduce a comprehensive technical framework for monitoring newborn resuscitation and explain how it was implemented in two different settings: Kampala, Uganda, a low-income setting, and Hanoi, Vietnam, a lower middle-income setting.

## Materials and equipment

This low-cost video recording setup for quality improvement in the delivery room includes:

a) Video cameras and video storage:
  • Cameras: HD 1080P Black Box AI-IP018 (Shenzhen Aishine Electronics) (Uganda).
    HD 1080P PIR Black Box Wi-Fi Security Camera AI-IP018 cameras (Shenzhen Aishine Electronics) (Vietnam).
    (HD-DV018 1080P Black box PIR Security Camera, AI (Shenzhen Aishine Electronics) (First used in Vietnam but later taken away).
  • Memory space: SD card (SanDisk, Western Digitals, Milpitas, CA, USA) 128 GB with space for up to 3.5 days of recording.
  • Hard drives, one for raw footage storage and one for cut version, 4 TB (Western Digital My Passport, Milpitas, CA, USA).
  • Homemade heat-proofed camera cases.
  • Software for cutting and mending data: Adobe Premiere Pro 64-bit version (Adobe Systems Inc., Adobe, San Jose, CA, USA).

b) Heart rate monitoring equipment: NeoBeat Newborn HR Meter (Laerdal, Stavanger, Norway).
c) iPad platform with software NeoTapAS for video analysis.
d) Statistical software R.
e) Local resuscitation table.
f) Hospital-based neonatal resuscitation guidelines.

In the list above, we used specific brands for practical quality reasons. However, similar equipment can be used.

## Methods

We describe a low-cost monitoring setup for the resuscitation table in the delivery room. This method helps to identify the process and actions of staff procedures to improve the quality of care. The method has been used at Mulago National Referral Hospital, Uganda, and Phu San Hanoi Hospital, Vietnam.

### Methodology setup

#### First step

We reviewed existing procedures and equipment, the organization of the resuscitation team, and current guidelines. This included discussions with the administration and clinical staff of the hospitals about how to minimize interference of monitoring with current practices and information about the study and equipment aimed to be used.

#### Second step

At both sites, ethical approval was sought from the local institutional review boards. The decision regarding the number of cameras and NeoBeat monitors needed for the studies was done according to where most neonatal resuscitations took place at the hospitals.

#### Third step

The equipment was installed to identify potential technical problems before the training of the staff. Cameras were placed at each resuscitation table away from the high-temperature area of the heater to capture exact footage of the newborn and the hands of the providers, eliminating the risk of staff identification. Also, placement was optimized for easy SD card access needed for the data collection. Tables were adapted to fit the cameras within the recommended focus range, between 60 and 120 cm. Cables and NeoBeat chargers (24) were also placed on the resuscitation table to facilitate access and minimize interference in normal clinical practice.

#### Fourth step

Midwives, doctors, nurses, and technical staff were introduced to the study setup and trained in equipment management. Hospital executives were informed about practice changes, and cleaning staff were trained to avoid tampering with the new setup.

#### Fifth step: clinical usage

##### Uganda

**Figure 1:**
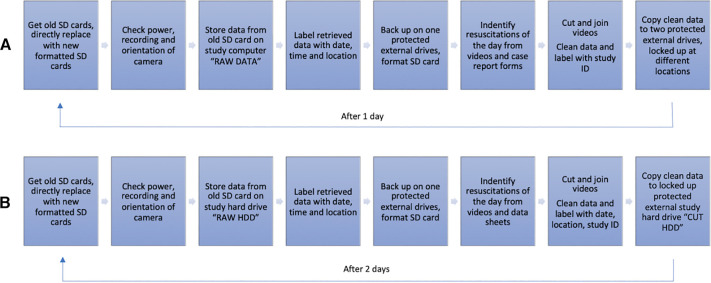
Video camera process for Uganda (**A**) and Vietnam (**B**) from the insertion of SD card into camera to video recording and final cut ready for analysis.

Video cameras HD 1080P Black Box AI-IP018 (Shenzhen Aishine Electronics) were installed at three out of four resuscitation tables at Mulago National Referral Hospital, Kampala, Uganda, and recorded data around the clock. The cameras were placed so that only the hands of the resuscitator and the neonates were recorded. The SD cards of each camera were swapped every day to ensure that all recordings were properly saved (Figure 1A). In case technical issues were noticed, the study coordinator or trial investigator was notified for corrective measures. NeoBeat heart rate meters were installed for a sub-study, and therefore only used for part of the newborns. It was placed directly around the neonates’ abdomen, providing a fast and accurate continuous display of heart rate visible on the videos during the review process (29). Inclusion criteria were neonates born in the hospital, estimated gestational age of at least 34 weeks, estimated birth weight of at least 2,000 g, and need of PPV at birth. Exclusion criteria were major malformations (incompatible with sustained life or affecting the airways) and stillbirths. All neonates fulfilling the eligibility criteria for each clinical study (please find the separate clinical criteria in references by Myrnerts Höök et al., Pejovic et al., and Larsson et al.) were filmed and enrolled (30, 31).

Each morning and afternoon, the camera data manager checked the cameras to ensure that they functioned well, including power control and the orientation of the camera. In case of problems, the study coordinator or trial investigator was notified for corrective measures. Power banks were used to keep the cameras running in case of a power shortage. The steps of retrieving and saving data are summarized in step six. After retrieving data, each resuscitation of the day was identified by the data managers and shown to one of the study doctors to ensure that it was a case of need of PPV and that the complete case was caught on video.

##### Vietnam

**Figure 2:**
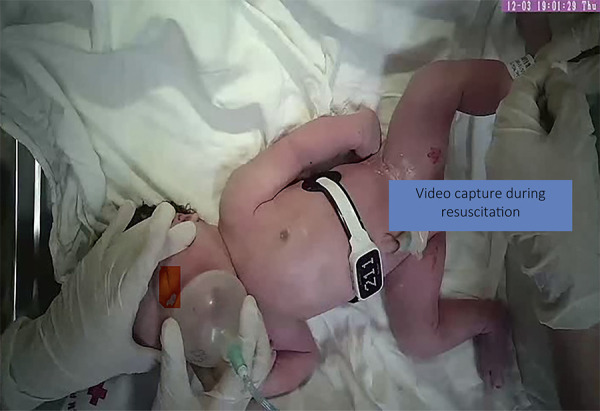
A real capture from a video showing the situation at the resuscitation table with NeoBeat on the neonate's chest and only hands shown.

**Figure 3:**
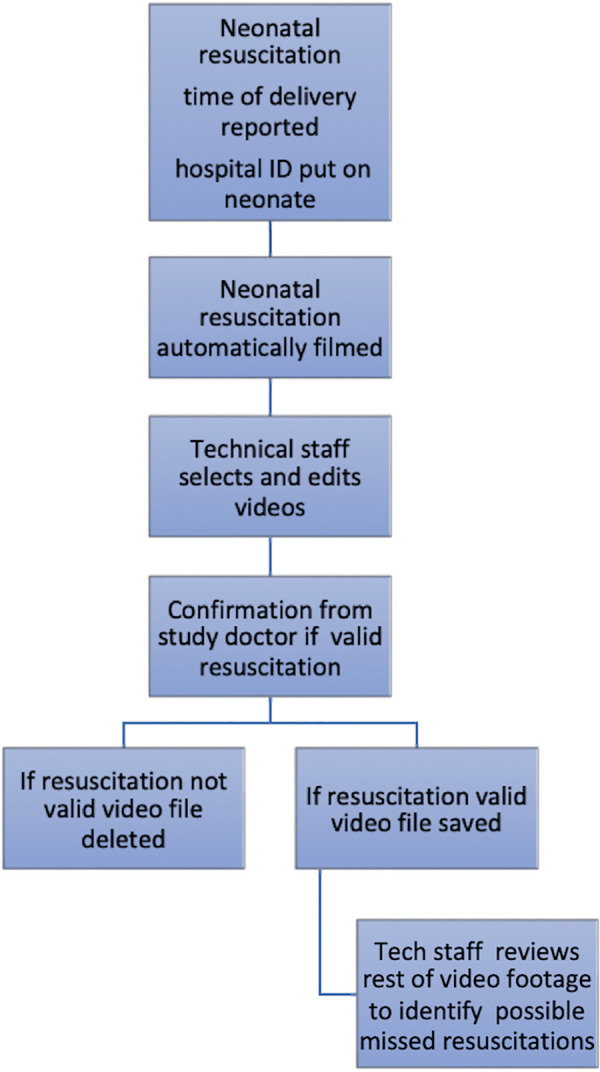
The process from delivery to verified video captured resuscitation, Uganda, and Vietnam.

Video cameras HD 1080P PIR Black Box Wi-Fi Security Camera AI-IP018 cameras (Shenzhen Aishine Electronics) (32) were installed above 7 out of 12 Phu San Hospital resuscitation tables, Hanoi, Vietnam, and recorded data around the clock. The cameras were placed so that only the hands of the resuscitator and the neonates were recorded (Figure 2). Inclusion criteria were born in a hospital, and need for PPV at birth. There were no exclusion criteria. The SD cards of each camera were swapped every second day (but the cards used could handle data up to 3.5 days) to ensure that all recordings were properly saved (Figures 1B). In case technical issues were noticed, the study coordinator or trial investigator was notified for corrective measures (Figure 3). NeoBeat heart rate meters were installed at each of the seven resuscitation tables (33), and used in the same way as above for the newborns with placement around the neonates’ abdomen, providing a fast and accurate continuous display of heart rate visible on the videos during the review process (33).

#### Sixth step: collection of data

Each camera’s footage was secured by swapping the SD card and checking that the camera was recording the correct angle. The SD card with the latest data was downloaded onto the first hard drive (4 TB, Western Digital My Passport Milpitas, CA, USA) and marked RAW, identifying the date, and camera. The data were stored in folders with formats as follows:

• RAW: Date -> Camera number -> Videos

The technical staff downloaded all video data including identified resuscitations. The time when the resuscitations took place was recorded by the attending midwife or doctor. The sections with important data were selected using Adobe Premiere 64-bit version and transferred to storage in a 4 TB hard drive. A final evaluation of the resuscitation recordings was performed by a study doctor, also checking for missing data. The videos were stored in folders with formats as follows:

• CUT: filename of videos (ORDERNO_DDMMYY_LOCATION_ CAMNUM_PATIENTID) Patient ID was a generated study number.

#### Seventh step: data management for analysis

Data extracted from the video recordings as well as maternal and neonatal characteristics were collected using standardized hard-copy case report forms. Resuscitation data were collected by analysis of the videos, using the NeoTapAS (34) app for real-time data registration during neonatal procedures (25, 26). Recorded data were directly exported for statistical analysis.

### Statistical methods

We used the free R statistical software [see previous papers (19, 33)]. The code documentation and scripts are available on request. Example scripts used to reproduce the figures have been published on GitHub were also the R package with functions and example data can be found. https://github.com/Global-child-health-SDGs-Team-KI/Neonatal-Resuscitation.

### Data management

At both study sites, all study-related information obtained was handled confidentially. All participant information, including case report forms, lists, logbooks, and any other listings linking participant ID numbers, was stored in locked file cabinets in areas with limited access. Research teams could only identify participants using a locally stored key file linked to the study numbers. Raw files were stored on a primary drive, cutout videos on a secondary drive, and backup copies were stored in a remote location (Figure 1). Videos were stored in a locked safe and hard drives were password protected. To help with the usage of the method, the collection types, variables used, and codes are available on request even if the real collected data due to ethical considerations cannot be shared.

### Ethical considerations

The protocol for the Ugandan study was approved by the Institutional Review Board of Mulago National Referral Hospital, Uganda (MHREC 1168), the Uganda National Council of Science and Technology; the Director-General from the Ministry of Health, Uganda (MREC 1168); and the Regional Committee for Medical and Health Research Ethics (REK South-East reference number 2017/989) in Norway. The protocol for the Vietnamese study was approved by the Institutional Review Board at Dinh Tien Hoang Institute of Medicine, the Ethical Review Board of Hanoi (No: IRB-2001 and IRB-2002), and the Swedish Ethical Review Authority (Dnr 2021-00064/2021-03-01).

Video recording with ethical considerations is today the standard for data collection in neonatal resuscitation (21, 35–37). Only the neonate and the hands of the resuscitator were visible, and the video audio was muted. The identity of neonates and resuscitators were kept anonymous. Video recordings were not used for malpractice cases. Thus, no consent was required from the resuscitators. All staff involved at both sites in neonatal resuscitations got at least one information session prior to study commencement. If the parents of the neonate did not consent to participate in the study, the video recordings were deleted and safely discarded.

## Results

The technical setup focused on neonatal resuscitation in two LMICs and had parallel protocols. We anticipated that the setups could produce data about the procedures and team performance in both LMICs. Below we summarize our experiences in the two different study settings.

### Uganda

Cameras were susceptible to overheating, and two cameras had to be replaced because of battery swelling. This happened although the resuscitation table heaters at Mulago Hospital were not in use. Power shortage was challenging but solved by connecting extra power banks to the cameras. We had cameras offline to avoid unsafe transferring data *via* Wi-Fi. The high quality of the camera footage allowed us to zoom in during the review, using standard software to identify additional details. A hanging kit using steel wires and duct tapes was made to secure the cameras. However, these hanging kits were difficult to reuse and magnetic adjustable camera mounts improved operations.

A total of 17,505 eligible neonates were born in the hospital during the study period of which 16,781 provided oral consent and 1,439 needed PPV. None were excluded because of missing video or poor quality. In total, 268 neonates were excluded due to other reasons. Thanks to the video reviews eight neonates were withdrawn from the main trial (four were fresh stillbirths, two did not need PPV, and two had major dysmorphic features) (31).

For handling case report form data collected simultaneously as video data, we used a Fujitsu iX1500 scanner to help us export the data for inclusion in our electronic database (38).

### Vietnam

**Figure 4:**
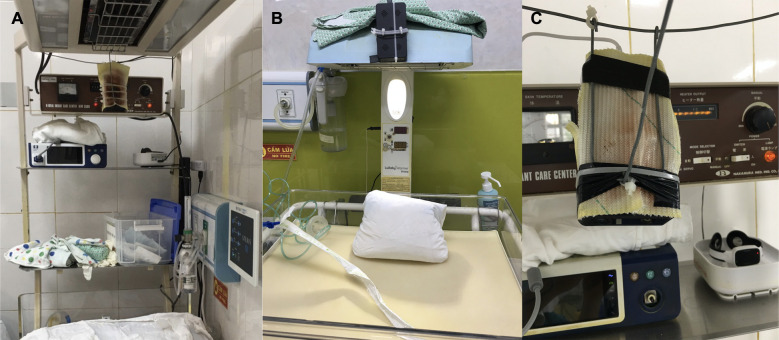
(**A**) The capture of setting up version 1. (**B**) The capture of setting up version 2. (**C**) Camera close-up.

To avoid overheating the cameras, we used three different setups. Staff surrounding the tables were trained to address possible overheating and avoid moving the camera angle (Figures 4A,B). In the most intensive resuscitation tables, we found two cameras that overheated, and the shell melted, causing another view angle than intended. The original chargers were prone to failure (the USB port and camera cable breaking or losing contact), but third-party chargers solved the problem.

**Figure 5:**
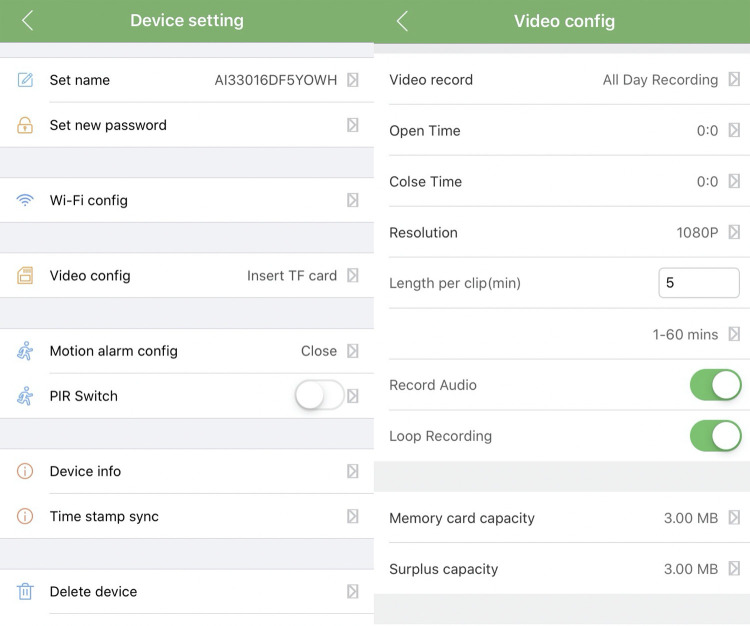
App software of the camera in Vietnam.

We initially used an HD-DV018 1080P Black box PIR Security Camera, AI (Shenzhen Aishine Electronics) (32). However, cameras with internal network HD 1080P Black Box AI-IP018 cameras (Shenzhen Aishine Electronics) and an app for IOS or Android (supposed to be fully functional but not fully tested by our team) for remote check improved trimming of camera angles. High-definition video data allowed us to identify additional details. The compatibility of the camera management application was limited as it only supported IOS for iPhones. The cameras did not have a hanging kit, so the installation of the cameras was made in a similar way as explained above for Uganda. The Software of the cameras was changed between models from pro version 1 to pro version 2. The new version improved stability during power cuts and program performance. However, problems with inaccurate time stamps on videos and shut-down operation of two-way audio recording still need to be addressed with software updates (Figure 5).

Midwives were unfamiliar with the new NeoBeat heart rate monitors (24) and needed training. The research teams' subjective view is that NeoBeats were found to be user-friendly, and both midwives and doctors found them helpful for clinical assessments. During the study, we also used NeoBeat for neonates that were smaller than recommendations, often with a continuously displayed heart rate despite a birth weight <1,500 g and using a plastic bag. We did not compare the displayed heart rate with heart auscultation but have no reason to mistrust its accuracy. NeoBeat has now become essential equipment during newborn resuscitation at the Phu San hospital.

A total of 18,107 eligible neonates were born in the hospital during the study period of which 75 needed PPV. Fifty-seven neonates who received PPV were captured on a video camera, of which all provided oral consent. Thirty-six neonates required endotracheal intubation of which 24 were captured on video camera. The reasons for the neonates that were not captured on video recordings were: resuscitation on location with no camera (main reason); a problem with the camera (broken, not charged). No videos had poor quality. No neonates were withdrawn from the study after the video review.

For handling case report form data collected simultaneously as video data, we used a Fujitsu iX1500 scanner to help us export the data for inclusion in our electronic database (38).

#### Documentation of neonatal resuscitation practices

The quality of the overall documentation of neonatal resuscitation in medical charts has been investigated and points to an urge to improve documentation to evaluate resuscitation practices (39). In Uganda, midwives were informed to record with a stopwatch, the time from the start of ventilation to the end when the resuscitation device was removed from the baby's mouth. Times were noted in the case report forms. When reviewing the videos, we saw that the ventilation times were inaccurate and could not use the data in the first published paper (31). In a recent sub-study (29), ventilation time was recorded by looking at the videos and recording the time of start and stop of ventilation. A comparison of ventilation time from the case report forms and this method is noted in Table 1. This points to the benefit of not just relying on on-site documentation by individuals for reliable data.

**Table 1:** Case report form vs. video review.

No	Ventilation time* noted in case report form in seconds	Ventilation time* according to video review in seconds
1	1,345	2,100
2	57	60
3	112	189
4	44	52
5	40	55
6	895	2,171
7	95	120
8	1,250	1,412
9	131	169
10	90	92
11	650	1,007
12	387	2,560
13	330	490
14	76	87
15	112	110
16	121	100
17	65	85
18	80	68
19	57	102
20	245	284
21	80	117
22	54	87
23	128	125
24	108	118
25	131	136
26	150	263
27	308	376
28	324	402
29	690	726
30	113	197
31	18	19
32	369	2,254
33	1,086	1,370
34	250	287
35	800	888
36	83	121
37	396	2,266
38	291	592
39	109	168
40	118	195
41	194	261
42	105	162
43	101	192
44	48	54
45	798	983
46	46	57
47	707	886
48	39	56

* Ventilation time is the time in seconds from the start of ventilation to the end when the resuscitation device is removed from the baby's mouth.

In summary, we collected detailed resuscitation video footage with data showing the procedures done by the staff hands at the resuscitation table, including stimulation, ventilation, intubation, and heart rate monitoring with NeoBeat. NeoTapAS allowed fast and accurate registration of all events during video review. All events were automatically summarized in Excel format in a report ready for export. The information could then be directly exported to other statistical programs, such as R, for further analysis. The video footage provided crucial information to improve the quality of care during neonatal resuscitation in the delivery room.

**Important lessons learned:**

–  Staff should be informed to notify data managers early if problems occur.
–  Each resuscitation table should have a charging station or extra batteries.
–  High-definition cameras with a minimum storage of 3.5 days are essential to eliminate the risk of running out of data storage if for unexpected reasons not changed according to plan.
–  In both settings, it got very hot at the resuscitation tables and the cameras were sensitive to heat, so we needed to protect the video cameras. In both LMIC settings, we have done this by optimizing/changing the placement of the camera and putting different kinds of heat shields around the most sensitive parts of the cameras and internal battery, as shown in Figures 4A–C.
–  NeoBeat charging routines and correct placement of the device on the baby’s abdomen are critical for accurate data collection.
–  Even if relatively low-cost and easy to install, selecting the resuscitation tables where the equipment should be installed is important. Each extra camera takes time and adds an extra burden.
–  Video analysis for data documentation during neonatal resuscitation is not a perfect alternative. It is sometimes difficult to assess the quality of ventilation and the depth of suctioning. It is never possible to see the endotracheal tube position. The heart rate and saturation monitor needs to be placed so that they can be viewed on the video recordings, and even if the NeoBeat heart rate monitor is used it can sometimes be difficult to see the digits due to light reflections.

If ethical approval allows, the video recordings can be used for training purposes and staff debriefing.

## Discussion

We describe a method for a low-cost video recording setup for quality improvement at the newborn resuscitation table. The technical framework, data collection, and analyses used in one low- and one LMIC for identifying resuscitation procedures can provide highly detailed resuscitation video footage including data on procedures and team performance, heart rate monitoring, and clinical assessment of the neonate.

Video recording for data documentation during neonatal resuscitation is increasingly regarded as a golden standard of practice. Both our own (19, 20, 29, 30) and other (6, 21, 22, 27, 35, 40) research teams have previously conducted similar studies. Usage of video recording, heart rate monitoring, and NeoTapAS in these studies were useful tools to identify performance at the resuscitation table (17, 19, 20, 28–31, 33, 41) and improve data collection. This method may be helpful for a better understanding of patients included in studies and treatment of hypoxic-ischemic encephalopathy (HIE) after resuscitation (41).

Video footage also provided objective feedback on staff practice and performance, helping them to improve practice regardless of the research outcome. Progress in clinical practice during data collection is a confounder from a research perspective but justified in the context of site-specific quality improvement programs. The technical setup also holds the potential to be used in telemedicine programs in the future.

We encountered technical issues such as overheating of cameras and software problems, but these issues were swiftly addressed. In recent studies, we used cameras with improved heat control, updated software (42), and higher resolution. These updates created a solid data collection setup and serve as an additive to the traditional setup used in Uganda and Vietnam to identify neonates in need of PPV. Video evaluation allows screening and documentation of cases of neonatal birth asphyxia in risk of HIE and staff debriefing. This setup could also contribute to aligning current hospital guidelines with best practice recommendations including improving the quality of neonatal resuscitations.

This low-cost video recording setup has been used to monitor neonatal resuscitation. However, this method has the potential to be applied within a range of different areas to improve upon skills, procedures, and learning from experience, e.g., emergency care and trauma care.

Video recordings can support staff debriefing and be embedded in site-specific quality improvement programs. The recordings can be used when training close-loop communication, in debriefings after complicated resuscitations with unpredicted results, and in general quality improvement programs focusing on neonatal resuscitation team training.

## Conclusions

Video analysis of neonatal resuscitation is an emerging quality assurance tool with the potential to improve neonatal resuscitation outcomes. The setup is well adapted for low- and lower-middle-income settings where improvement of neonatal resuscitation outcomes is crucial. The cameras also support two-way audio communication and could potentially be used with remote tele leaders or teleconsultants in the future.

## Data Availability

The datasets presented in this study can be found in online repositories. The names of the repository/repositories and accession number(s) can be found at: https://github.com/Global-child-health-SDGs-Team-KI/Neonatal-Resuscitation.
